# Editorial: On poverty and its eradication

**DOI:** 10.3389/fsoc.2024.1487220

**Published:** 2024-10-01

**Authors:** Andrzej Klimczuk, Guillermina Jasso, Mariah D. R. Evans, Jonathan Kelley

**Affiliations:** ^1^Department of Social Policy, Collegium of Socio-Economics, SGH Warsaw School of Economics, Warsaw, Poland; ^2^Department of Sociology, New York University, New York, NY, United States; ^3^Department of Sociology and Applied Statistics Program, Nevada Agricultural Experiment Station, University of Nevada, Reno, Reno, NV, United States; ^4^Department of Sociology, University of Nevada, Reno, Reno, NV, United States

**Keywords:** poverty, poverty relief, poverty and inequalities, poverty and fairness, poverty and public policy

## Overview

The Research Topic “*On poverty and its eradication*” was inspired by the International Day for the Eradication of Poverty, first commemorated in Paris in 1987 and formally designated by the United Nations. This day is dedicated to renewing the commitment to universal human development, enabling all individuals to achieve their highest potential, and reflecting on how poverty hinders this progress. The urgency of addressing poverty has increased after the COVID-19 pandemic, which exacerbated existing issues and highlighted the critical need for effective poverty eradication strategies. The studies included in this Research Topic aim to expand knowledge about poverty, its causes, consequences, and connections to crises, inequality, and fairness, and to assess policy measures for poverty reduction. The included contributions use various theoretical, empirical, quantitative, and qualitative approaches to drive sociological and policy-related discussions and initiatives, promoting fair and inclusive societies.

The presented Research Topic includes seven articles written by 29 authors from the following countries: China, the Philippines, the United Kingdom, and the United States. This Research Topic was co-organized in collaboration with five journals: “Frontiers in Human Dynamics,” “Frontiers in Political Science,” “Frontiers in Psychology,” “Frontiers in Public Health,” and “Frontiers in Sociology.” The studies comprising this Research Topic are divided into four themes.

## Theme I: entrepreneurship and consumption in poverty alleviation activities

In the context of this Research Topic, Valle et al. focused on studying the actions undertaken by higher education institutions to help reduce poverty by implementing community extension programs. Such schemes may focus on capacity building and entrepreneurship training. The analysis assessing these programs found that entrepreneurship education, budgeting, financial literacy, and access to credit facilities contributed to poverty reduction. However, entrepreneurial performance did not show a significant direct impact due to the COVID-19 pandemic crisis. Despite these challenges, the findings highlight the importance of educational and financial support in promoting entrepreneurial success and alleviating poverty. Xin et al. presented different approaches to combating poverty focused on consumption-support measures. The authors argued that charity, while effective in helping vulnerable groups, is often short-term and creates dependency, leading to adverse effects. Thus, the authors analyzed China's poverty alleviation model through “altruistic consumption,” which offers a sustainable alternative by leveraging pro-social behavior to support groups in need. The study found that altruistic consumption is driven by motivations related to group benefit, morality, demanders, and suppliers, with benefit-demander motivation having the most significant influence. These insights can guide targeted marketing strategies to sustain altruistic consumption efforts.

## Theme II: digital growth in poverty reduction

Two studies focused on the use of e-commerce platforms to alleviate poverty. Li et al. continued the discussion on consumption-based poverty reduction in China. The authors showed that this approach has a crucial role in rural revitalization, but its sustainability is challenged by low consumer participation and reliance on government procurement. Trust is a significant factor affecting consumers' willingness to buy “poverty-alleviation agricultural products,” primarily due to past adverse events that have damaged trust, such as slow sales. The study found that enhancing trust through, among others, user feedback mechanisms, platform supervision, product traceability, and certification mechanisms can increase consumers' purchase intentions and promote sustainable rural development. Wu et al. went into further detail on the potential of e-commerce systems. Based on collective efficacy and risk perception theories, their analysis found that symbolic ethical attributes positively influence consumers' willingness to buy products online more than their functional attributes. The research highlighted the mediating roles of collective efficacy and risk perception, offering practical suggestions for e-commerce enterprises and marketers to enhance their farmer-assisting marketing strategies.

## Theme III: the role of health and employment in poverty mitigation

Another important trend observed in the papers included in this Research Topic is the combination of poverty alleviation measures with public health policies. Hou et al. presented a study that explored the impact of the New Rural Cooperative Medical Scheme (NRCMS) on the health status of the relatively poor population in rural China, using data from the China Family Panel Studies. Employing propensity score matching methods, the analysis found that participation in the NRCMS significantly improved the health status of low-income individuals. The positive health effects were attributed to increased physical activity, more frequent medical care visits, and reduced healthcare expenditures. Using a different perspective, Wang et al. focused on disability as a significant contributor to global poverty. The authors studied how China's welfare reforms and employment interventions have been implemented to address this issue. By employing the Alkire-Foster method, the researchers measured multidimensional poverty among subjects with disabilities aged 16–59 in China, finding that a high percentage were deprived in at least one dimension, particularly education and social participation. Employment services were shown to significantly reduce multidimensional poverty across various fields, providing crucial evidence to inform public policies aimed at eradicating poverty among people with disabilities.

## Theme IV: poverty and the income tax system

The final section includes a study by Jasso that argued that the linear income tax system, which aligns with principles of tax equity by increasing post-tax income, the amount of tax, and the rate of tax as pre-tax income increases, is an effective tool for poverty reduction. The study highlighted a potential issue where policies that help the poorest may harm the middle class, thus weakening this crucial segment of society. Moreover, the article contrasted different income distributions and demonstrated how to maintain tax fairness, even with additional resource injections, and how to estimate fair parameters for the linear income system.

## Conclusion

The results presented in the articles in this Research Topic allow the articulation of six directions for further research. These are: (1) new understandings of poverty, absolute and relative poverty (see Brady and Burton, 2016; Greve, 2020); (2) new approaches to the measurement of poverty (see Beck et al., 2020; UNECE, 2017; Mysíková, 2021); (3) causes, effects, and forms of poverty, in the contemporary world and historically (see Curtis and Cosgrove, 2021); (4) interactions between poverty and situations of crisis and disasters such as pandemics and conflicts (see Fitzpatrick, 2019; Greve, 2021); (5) new links between poverty and inequality (see Edward and Sumner, 2019; UNRISD, 2022); and (6) national and international policies and programs to eradicate poverty (see Wang et al., 2020; Oberholzer, 2022).
